# Structural variants and tandem repeats in the founder individuals of four F_2_ pig crosses and implications to F_2_ GWAS results

**DOI:** 10.1186/s12864-022-08716-0

**Published:** 2022-09-03

**Authors:** Iulia Blaj, Jens Tetens, Jörn Bennewitz, Georg Thaller, Clemens Falker-Gieske

**Affiliations:** 1 Institute of Animal Breeding and Husbandry, Kiel University, Kiel, Germany; 2 Department of Animal Sciences, Georg-August-University, Göttingen, Germany; 3 Center for Integrated Breeding Research, Georg-August-University, Göttingen, Germany; 4 Institute of Animal Husbandry and Breeding, University of Hohenheim, Stuttgart, Germany

**Keywords:** Structural variants, Tandem repeats, Genome wide association studies, Imputation, Pig, Whole-genome sequencing, lncRNA

## Abstract

**Background:**

Structural variants and tandem repeats are relevant sources of genomic variation that are not routinely analyzed in genome wide association studies mainly due to challenging identification and genotyping. Here, we profiled these variants via state-of-the-art strategies in the founder animals of four F_2_ pig crosses using whole-genome sequence data (20x coverage). The variants were compared at a founder level with the commonly screened SNPs and small indels. At the F_2_ level, we carried out an association study using imputed structural variants and tandem repeats with four growth and carcass traits followed by a comparison with a previously conducted SNPs and small indels based association study.

**Results:**

A total of 13,201 high confidence structural variants and 103,730 polymorphic tandem repeats (with a repeat length of 2-20 bp) were profiled in the founders. We observed a moderate to high (*r* from 0.48 to 0.57) level of co-localization between SNPs or small indels and structural variants or tandem repeats. In the association step 56.56% of the significant variants were not in high LD with significantly associated SNPs and small indels identified for the same traits in the earlier study and thus presumably not tagged in case of a standard association study. For the four growth and carcass traits investigated, many of the already proposed candidate genes in our previous studies were confirmed and additional ones were identified. Interestingly, a common pattern on how structural variants or tandem repeats regulate the phenotypic traits emerged. Many of the significant variants were embedded or nearby long non-coding RNAs drawing attention to their functional importance. Through which specific mechanisms the identified long non-coding RNAs and their associated structural variants or tandem repeats contribute to quantitative trait variation will need further investigation.

**Conclusions:**

The current study provides insights into the characteristics of structural variants and tandem repeats and their role in association studies. A systematic incorporation of these variants into genome wide association studies is advised. While not of immediate interest for genomic prediction purposes, this will be particularly beneficial for elucidating biological mechanisms driving the complex trait variation.

**Supplementary Information:**

The online version contains supplementary material available at 10.1186/s12864-022-08716-0.

## Background

Genome wide association studies (GWAS) aim to identify associations between genotypes and phenotypes. The term “genotype” commonly refers to SNPs, as most GWAS are performed using data from SNP arrays, primarily due to cost efficiency, high-throughput, and stability of the SNPs. With a steady increase in the volume of whole-genome sequence (WGS) data, within the variant discovery step, alongside SNPs, small insertions and deletions (indels; < 50 bp) can be similarly called and incorporated in the GWAS. Although further variation classes exist, such as structural variants (SVs) and tandem repeats (TRs), they are not considered in current association studies because they are not routinely screened. However, in the light of high-depth WGS data, it is now feasible to profile a wider spectrum of variation provided that appropriate algorithmic approaches exist [9, 11, 34, 54, 62]. Thus, these can be employed to capture a wide range of variant sizes and subclasses of SVs and TRs.

Structural variants are large genomic alterations, extremely diverse in type and size that can be typically classified as deletions, insertions, duplications, inversions, and translocations and can generally be characterized by various combinations of DNA gains, losses, or rearrangements [31]. To date, a limited number of studies cover SVs related investigations in pigs, for example in relation to selection signature identification in Meishan [18] or to associating Copy Number Variation regions (CNV; a particular subtype of SV) with growth and fatness traits in Duroc [57]. Besides SVs, tandem repeats are an additional type of sequence variation. TRs can be divided into short tandem repeats (STRs or microsatellites) with a core motif of 2 to 6 bp and variable number tandem repeats (VNTRs) with core motifs larger than 7 bp [30]. The primary driver for TR expansion or contraction is the polymerase slippage during DNA replication that also leads to an extremely high mutation rate [21]. Given their high degree of genetic variability, TRs often display high levels of heterozygosity and a multi-allelic nature. This aspect was initially viewed as an advantage making the TRs (namely the microsatellite) *the* standard genetic marker. However, subsequently it was seen as a disadvantage, because compared with the emerging SNPs, TRs were rather unstable and challenging for high-throughput screening. Regardless of their faith over time, the TRs are large contributors to the overall genetic variation. There is a growing body of evidence suggesting that TRs play a critical role in the regulation of gene expression [29] and splicing [33], as well as via DNA methylation [58]. Specifically for the pig, there have been efforts made to characterize TRs using WGS data with a focus on STRs [45, 72].

In this study, to investigate the genome-wide structural variants and tandem repeats landscape in pigs, we first profiled such variation across the founder generation of four F_2_ pig crosses originating from various breeds (i.e. Piétrain, Landrace, Large White, Meishan and Wild boar). We employed state-of-the-art detection strategies to screen SVs and TRs relying on high coverage WGS data. Further, we examined this variation in contrast with the commonly addressed polymorphisms (SNPs and small indels) in terms of density and genome localization but also functional impact. We imputed the SVs and the TRs information to the F_2_ generation and together with phenotypic data on average daily gain (ADG), backfat thickness (BFT), meat to fat ratio (MFR), and carcass length (CRCL) we conducted a SVs and TRs based GWAS. Therefore, in a second step, we evaluated the implications that SVs and TRs have for GWAS and devised how this particular type of variation aids to gain deeper insights into the genetic basis of complex traits.

## Results

### Discovery phase

The first part of this study focused on providing a sequence-based systematic characterization of the different types of variation (i.e. SNPs, small Indels, SVs, and TRs) existing in the founder individuals (*n*=24, Table SM1) of four F2 experimental crosses. The number of the various variants was proportional to the length of the chromosomes. An overview of the chromosome-wise distribution of all the variation identified is displayed in Table 1.

**Table 1:** Number of variants per chromosome

**SSC**	**SNPs**	**Small indels**	**Structural variants**	**Tandem repeats**	**Polymorphic tandem repeats**
**1**	2,100,792	492,397	1,268	104,628	10,893
**2**	1,447,573	343,180	908	56,192	6,574
**3**	1,386,966	291,597	716	49,565	5,897
**4**	1,342,424	290,615	765	49,228	5,929
**5**	1,157,084	267,350	714	37,884	4,988
**6**	1,631,542	370,136	896	64,079	7,295
**7**	1,255,167	284,413	769	44,794	5,424
**8**	1,422,204	338,937	798	52,268	6,549
**9**	1,423,977	328,226	926	50,282	6,340
**10**	1,002,412	222,468	537	23,164	5,613
**11**	955,612	214,708	603	28,679	4,183
**12**	771,362	170,298	462	21,773	3,062
**13**	1,617,902	410,438	956	81,922	8,385
**14**	1,374,393	321,456	812	51,769	6,160
**15**	1,210,587	298,692	757	55,135	5,892
**16**	912,732	210,198	571	29,850	4,852
**17**	788,319	174,886	484	22,770	3,139
**18**	603,633	123,587	259	20,574	2,555
**Total**	22,404,681	5,153,582	13,201	844,556	103,730

The SNPs and small indels were profiled in our previous imputed-sequence based GWAS study [22] and they amounted to 22,404,681 SNPs and 5,153,582 small indels (< 50 bp). To obtain a thorough characterization of the variation in the founders, we considered further types of variants, i.e. SVs and TRs. A reliable detection of SVs, where each call was supported by three different callers (smoove, DELLY, and manta), led to a total of 60,669 SVs. After stringent filtering, the final call set contained 13,201 SVs from which 11,954, 1,080, 164, and 3 were deletions, duplications, translocations, and inversions, respectively. The cumulated length of the structural variants amounted to approximately 68 Mb representing 3% of the autosomal genome. The size of the structural variants ranged from 51 bp to 991,370 bp with 83.85% of them being shorter than 1000 bp (Table SM2). When looking at the length distribution of deletions as the most abundant type of SV, we observed a peak in the elements with a size of 250 bp up to 450 bp (Figure SM1). These SVs originate from retrotransposition and, due to their size, they can be identified as short interspersed nuclear elements (SINEs) which are known to occupy up to 10% of mammalian genomes [13]. Concerning the individual animal SV genome-wide zygosity levels, the highest number of reference homozygous variants belonged to the Wild boar (European) individual, whereas the highest number of heterozygous variants was found in the Meishan (Asian breed) (Figure SM2), directly reflecting its genetic distance from the reference genome (Duroc, a European breed). Worth mentioning is the fact that the high genotyping rate (threshold 0.8) set up for the SVs favored the common SVs existing in the European breeds. However, attaining such a stringency was required for the second part of the study (i.e. GWAS).

The TRs reference panel consisted of 1,462,304 variants with a motif length from 2 bp to 20 bp of which 83.49% belonged to the short tandem repeats group (2-6 bp). The tetranucleotide repeats were the most abundant (27.93%), followed by the dinucleotide repeats (16.39%) (Figure SM3). Given the cumulated length of 35.35 Mb, the library of TRs covered 1.56% of the autosomal reference genome, which was in line with previous reports [45, 72]. In the founder data set, after genotype and sample-specific filters, we retained 844,558 high-quality TRs calls of which 103,730 loci had non-reference alleles, further denoted as pTR (polymorphic TR). Among the pTRs, the number of variants profiled decreased with the period length, except for the tetranucleotide class. The allelic configuration of the repeats comprised loci with a number of two alleles and up to eleven alleles. We observed that an increase in the period length led to a decrease in the number of alleles (Table SM3) indicating that higher allelic variability is less common in repeats with longer motifs. However, the profiling of longer motif variants could be hindered by factors such as current TRs detection methods, sequencing depth, and sequence read length. From the pTRs genotyped 67,538 variants were polymorphic in more than 2 breeds, while 13,074, 10,783, 10,237, and 2,125 were TRs limited to the Piétrain group, to the Large White x Landrace and Large White group, to the Meishan individual and to the Wild boar individual, respectively.

### Density and co-localization of features

The distribution of the genomic features was assessed in 500 kb windows and can be visualized in the SM for SNPs, small indels, SVs, TRs, and pTRs (Figure SM4). Addressed in a pair-wise manner, we measured the strength of the correlations between the feature occurrences in 500 kb non-overlapping windows (Table 2). The highest degree of positive correlation (*r* = 0.87) was found when comparing the density of the SNPs with the one of the small indels, both types of variation being called via the same pipeline. Moreover, a mild positive correlation between SVs and SNPs (*r* = 0.48) or small indels (*r* = 0.49) was detected. When assessing the co-localization of the genotyped TRs with either SNPs, small indels, and SVs we observe a low to moderate negative correlation suggesting that the TRs occupy genomic regions in which other types of features are less prominent. However, when looking specifically at the pTRs, a contrasting scenario reveals a higher level of co-localization with the SNPs and small indels.

**Table 2:** Co-localization of different types of variation. Pearson correlation coefficient *r*-values based on 500 kb windows in autosomes are shown in the upper triangle; *p*-values are shown in the lower triangle

	**SNPs**	**Small indels**	**SVs**	**TRs**	**pTRs**
**SNPs**		0.87	0.48	-0.43	0.57
**Small indels**	*p* < 2.2e-16		0.49	-0.43	0.55
**SVs**	*p* < 2.2e-16	*p* < 2.2e-16		-0.23	0.36
**TRs**	*p* < 2.2e-16	*p* < 2.2e-16	*p* < 2.2e-16		0.01
**pTRs**	*p* < 2.2e-16	*p* < 2.2e-16	*p* < 2.2e-16	*p* < 0.37	

### Feature annotation and gene enrichment analysis

**Fig. 1:**
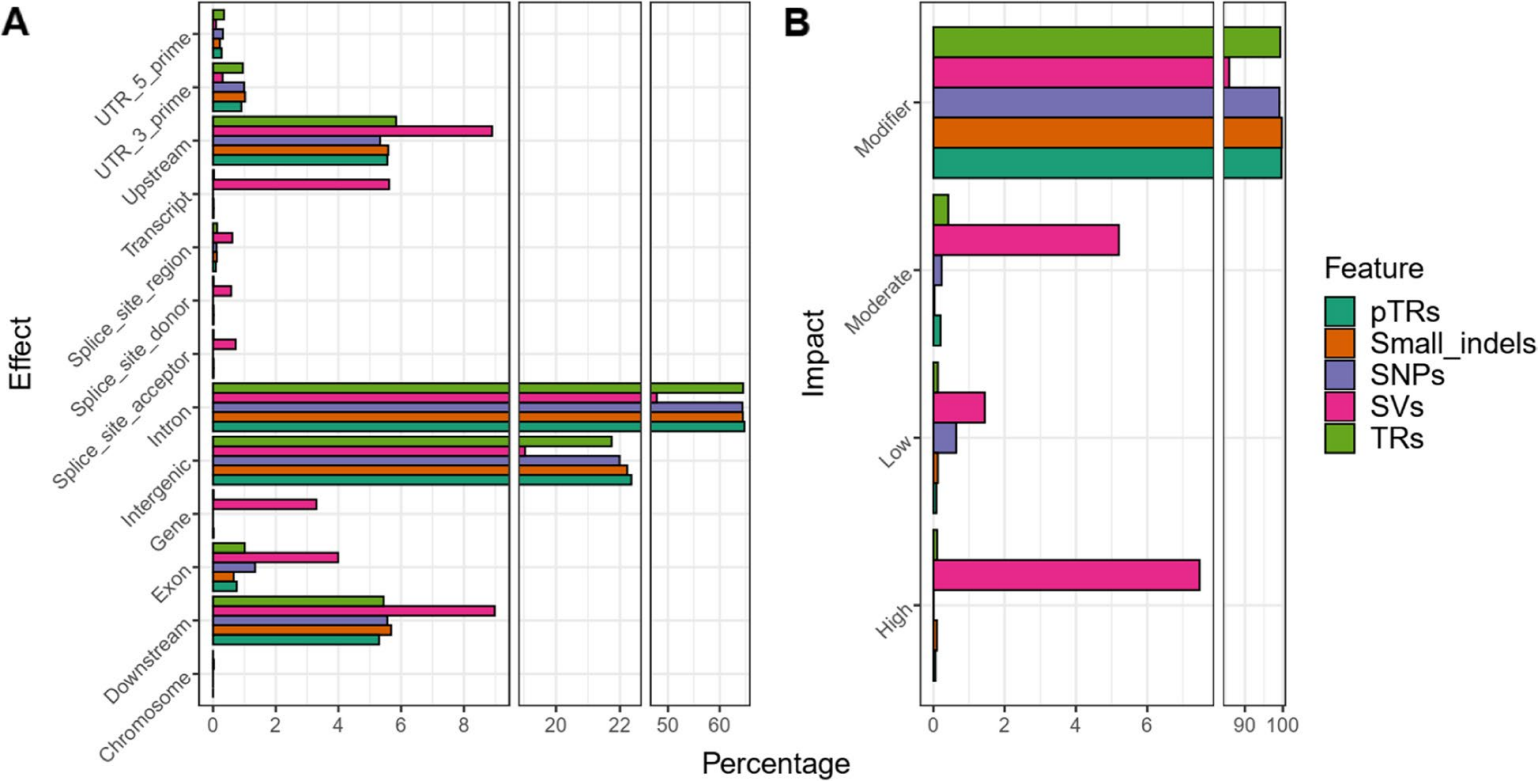
Variant annotation and impact classification. Percentage breakdown for the SNPs, small indels, SVs, TRs, and pTRs. **A** Percentage of effects classified by region for each type of variation. **B** Percentage of effects classified by impact for each type of variation

The number of functional annotations was 48,685,675, 13,725,269, 37,278, 492,658 and 324,331 for SNPs, small indels, SVs, TRs and pTRs, respectively. The percentage breakdown of the effects by region and by impact for each feature type is summarized in Fig. 1. The distribution of the variants across different genomic regions was similar for SNPs, small indels, TRs, and pTRs. However, the SVs displayed either smaller percentages in case of intronic (47.85%) and intergenic (19.03%) or higher percentages for downstream (8.98%), exon (3.99%), gene (3.30%), transcript (5.62%) and upstream (8.90%) as compared with the other feature groups. This aspect was also reflected when assessing the number of effects classified by impact type. The impact rating informs about the severity of a predicted consequence for a variant on a transcript or on a protein. The modifier effect was, as expected, predominant and displayed in more than 99% of the annotated SNPs, small indels, TRs, and pTRs. Nevertheless, in the case of the SVs, modifier effects only represented 85.88% of the total, whereas a share of 7.47% were high impact variants (i.e. the variant is assumed to have a disruptive impact on the protein, for example a stop gained or a frameshift type of variant).

We further prioritized on high, moderate, and low impact variants and, based on the overlapping genes, we defined gene sets for each feature to identify over-represented GO Biological Processes terms. Among the feature gene sets, we found 25 genes in common (Table SM4). In the case of the gene sets based on SNPs (*n*=19,483) and small indels (*n*=8,512), the GO enrichment analysis revealed that more than 500 GO terms passed the FDR threshold (0.05) and, in both cases, the most significantly enriched term was Localization (GO: 0051179) (Figure SM5). The rather large gene sets for the above-mentioned features are a direct consequence of the higher density of these elements along the genome and this is further reflected in the top enriched terms which are pinpointing to fundamental or basic biological processes (e.g. development and metabolism related terms).

**Fig. 2:**
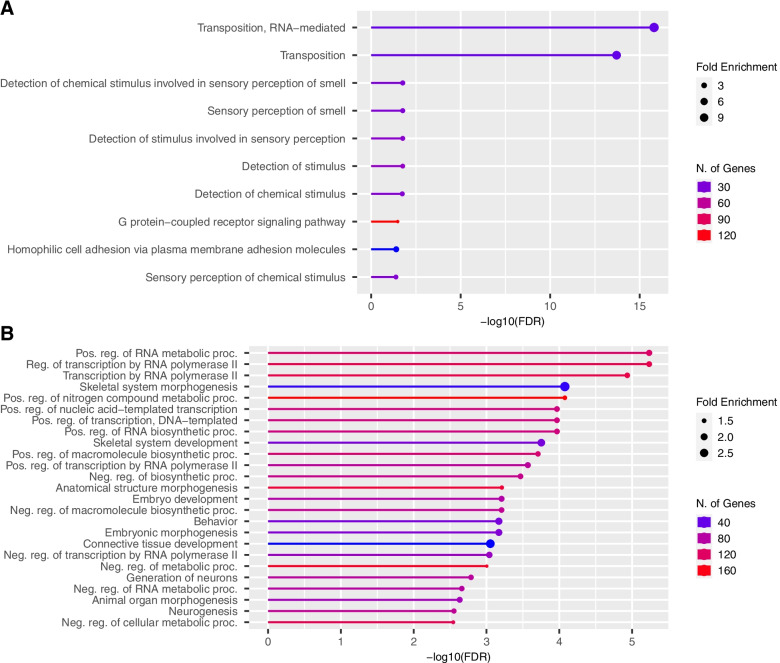
Gene enrichment analysis. **A**. Enriched GO Biological Processes for SVs overlapping genes (*n*=1,448); **B**. Top 30 enriched GO Biological Processes for TRs overlapping genes (*n*=1,229)

The gene enrichment analysis for the SVs gene set (*n*=1,448) revealed ten significantly overrepresented GO terms (Fig. 2A) related to transposition (GO: 0032197 and GO: 0032196), homophilic cell adhesion (GO: 0007156), and sensory perception of smell (e.g. GO: 0007608 and GO: 0050911). The enrichment in the transposition term was an effect of the high numbers of SINEs within the deletions group. Furthermore, the SVs appeared to be overlapping with a high number of olfactory receptors that led to having 7 out of 10 GO related terms enriched. This particular gene family is known to have a significant expansion throughout time as, based on the previous reference genome, it includes 1,113 functional genes and 188 pseudogenes [55]. The TRs gene set (*n*=1,229) yielded 146 enriched GO terms with the top 30 being depicted in Fig. 2B. Frequent GO terms belong to biological processes involved in various types of regulation (e.g. of transcription, metabolic or biosynthetic processes) indicating either the presence of TRs in regulatory regions and/or the influential role of the TRs in regulating gene expression. Finally, for the pTR gene set (*n*=445) no significant enrichment was found.

### GWAS results

**Fig. 3:**
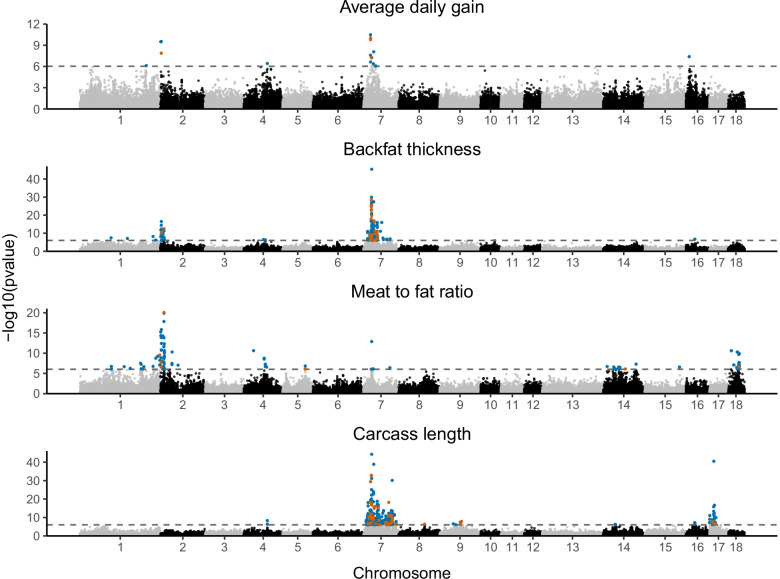
Manhattan plots of the genome wide association studies for the four traits. The genome-wide significant threshold is given by –log10 (0.05/54,075). Significant SVs are red, while significant TRs are marked by the color blue

From the genome wide association study, the following cumulated number of SVs and TRs exceeded the genome-wide significance threshold: 17, 156, 105, and 339 for ADG, BFT, MFR, and CRCL, respectively. For this step, 54,704 imputed SVs and TRs were tested for association. Manhattan plots for the four phenotypic traits are shown in Fig. 3.

**Fig. 4:**
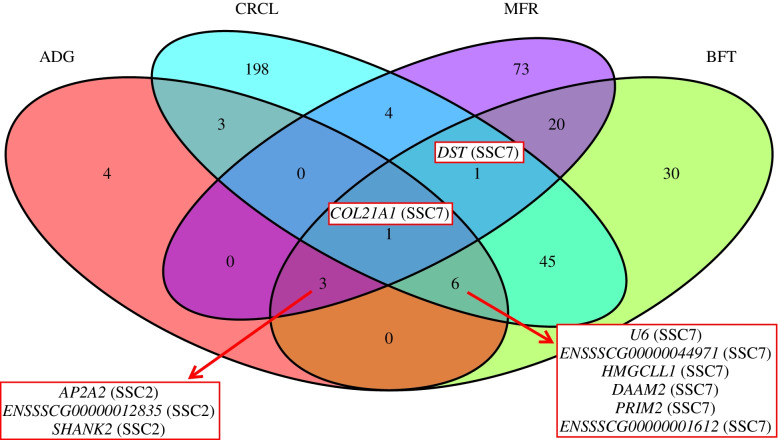
Venn diagram displaying all possible relations among the gene sets overlapping or in the vicinity of significantly associated SVs and TRs. These variants were not tagged (r^2^ < 0.8) by previously identified SNPs or small indels

The quantile-quantile plots were generated for all *p*-values from each GWAS and are reported in Figure SM6 together with the genomic inflation factor. The moderate degree of *p*-values inflation is attributed to the fact the “leave one chromosome out” analysis was used. As compared to our previous GWAS study [22] that relied on SNPs and small indels, the current study detected additional significant variants on SSC1 and SSC16 for ADG, on SSC16 for BFT, on SSC14 and SSC15 for MFR, and on SSC4, SSC8, SSC9, SSC14 and SSC16 for CRCL. On chromosomes with significant variants in both studies, SVs and TRs were subsequently selected based on LD. Therefore, in this post-GWAS analysis, we discarded all significant SVs and TRs that were in high LD (r^2^ > 0.8) with the previously found significant SNPs and small indels. The number of SVs and TRs that were not tagged by a significant SNP or small indel were 12 (out of 17), 112 (out of 156), 89 (out of 105), and 237 (out of 339) for ADG, BFT, MFR, and CRCL, respectively. From these variants, the top variants per chromosome were retained. The top five genes incorporating or lying in the proximity of these highly significant associations are presented in Table 3. Further, by including the top significant TR ((TTTG)_3_/(TTTG)_5_, SSC7:29,488,854) as a fixed effect in the LOCO mixed linear model, the significant signal dropped greatly for all traits, even below the designated threshold for ADG and MFR (Figure SM7, QQ plots in Figure SM8). To evaluate all possible relations among the gene lists overlapping or in the vicinity of the subsetted significant SVs and TRs for each trait, a Venn diagram was used (Fig. 4).

**Table 3:** Top five associated genes per chromosome for each trait: average daily gain (ADG), backfat thickness (BFT), meat to fat ratio (MFR), and carcass length (CRCL). Gene type in brackets. The variants overlapping or in the proximity of these genes were selected not to be in high LD (r^2^ < 0.8) with the previously associated SNPs and small indels

	**SSC**	**Top five genes**
**ADG**	1	*ZFAND5* (protein coding), *TMC1* (protein coding)
	2	*AP2A2* (protein coding), *ENSSSCG00000012835* (pseudogene)
	4	*UBE2V2* (protein coding)
	7	*U6* (snRNA), *ENSSSCG00000044971* (lncRNA), *HMGCLL1* (protein coding), *PRIM2* (protein coding), *COL21A1* (protein coding)
	16	*ENSSSCG00000050375* (protein coding), *CDH10* (protein coding)
**BFT**	1	*ENSSSCG00000048964* (lncRNA), *ENSSSCG00000043016* (lncRNA), *ENSSSCG00000051116* (lncRNA), *TXNL1* (protein coding), *ENSSSCG00000043998* (protein coding)
	2	*SHANK2* (protein coding), *STX3* (protein coding), *TNNT3* (protein coding), *CBLIF* (protein coding), *MRPL16* (protein coding)
	4	*PREX2* (protein coding),* CHD7* (protein coding)
	7	*COL21A1* (protein coding), *DST* (protein coding), *ENSSSCG00000001612 *(protein coding), *PRIM2* (protein coding), *ENSSSCG00000041331* (processed pseudogene)
	16	*ENSSSCG00000050819* (lncRNA), *ISL1* (protein coding)
**MFR**	1	*PLPP7* (protein coding), *PRRC2B* (protein coding), *RALGPS1* (protein coding), *RABGAP1* (protein coding), *ASTN2* (protein coding)
	2	*STX3* (protein coding), *CBLIF* (protein coding), *MRPL16* (protein coding), *SHANK2* (protein coding), *TNNT3* (protein coding)
	4	*ENSSSCG00000051273* (lncRNA), *LRP12* (protein coding), *CHD7* (protein coding), *RAB2A* (protein coding), *RGS20* (protein coding)
	5	*VDR* (protein coding), *RPAP3* (protein coding)
	7	*COL21A1* (protein coding), *ENSSSCG00000002296* (protein coding), *VTI1B* (protein coding), *KIF6* (protein coding), *ENSSSCG00000031184* (protein coding)
	14	*BTRC* (protein coding), *XKR6* (protein coding), *ENSSSCG00000045361* (lncRNA), *ENSSSCG00000051786 *(lncRNA), *ENSSSCG00000051722* (protein coding)
	15	*IGFBP5* (protein coding), *TNP1* (protein coding)
	18	*HERPUD2* (protein coding), *ENSSSCG00000042613* (lncRNA), *AOAH* (protein coding), *KIAA0895* (protein coding), *SND1* (protein coding)
**CRCL**	4	*UBE2V2* (protein coding)
	7	*COL21A1 *(protein coding), *HMGCLL1* (protein coding), *DAAM2* (protein coding), *ZNF451* (protein coding), *ENSSSCG00000044971* (lncRNA)
	8	*NOCT* (protein coding), *ENSSSCG00000049816* (lncRNA)
	9	*PON2* (protein coding), *ASB4* (protein coding), *ENSSSCG00000049806* (lncRNA), *NECTIN1* (protein coding), *ENSSSCG00000044947* (lncRNA)
	14	*ANKRD13A* (protein coding), *SPPL3* (protein coding), *WSCD2* (protein coding), *SGSM1* (protein coding)
	16	*ENSSSCG00000050819* (lncRNA), *ISL1* (protein coding)
	17	*ENSSSCG00000044805* (lncRNA), *PLCB4* (protein coding), *ENSSSCG00000043546* (protein coding), *ENSSSCG00000047884* (lncRNA), *PLCB1* (protein coding)

## Discussion

In the current study, we investigated structural variants and tandem repeats as a considerably less exploited resource of genomic variants with a special focus on their meaning in the context of genome wide association studies. To address this, we relied on data from four F_2_ pig populations and from a previous SNPs and small indels based GWAS [22]. In the F_0_ generation (WGS data, 20x), we had access to SNPs, small indels, SVs, and TRs. These variation classes varied in terms of their genomic properties including size, distribution across the genome, abundance as well as functional impact on the nucleotide sequence.

SVs occurred at a much lower frequency than any other type of variation, yet, concerning their functional annotation, they could potentially have a pronounced phenotypic impact by disrupting gene function (Fig. 1). These types of phenotypic consequences of the SVs have been observed in livestock species often in relation to pigmentation and coat color (cattle [19]; pig [63]), fertility (cattle [37]; pig [44]) or late feathering in chicken [20]. Initially, the effects of SVs (similarly to TRs) have been assumed particularly negative, partly due to the identification of many SVs (and TRs) associated with human disease [25, 70]. Despite this, many SVs are of a neutral or adaptive nature [3], positioning them as important evolutionary drivers. Our SVs panel was mainly formed by deletions (90.55%) identified by three callers (i.e. smoove, DELLY, and manta) suggesting that this particular type of SV was easier to detect by different algorithms [46]. In terms of mechanisms leading to deletion formation, transposable element insertions involving mostly short interspersed elements (SINEs) were greatly responsible [13] which we observed as an increase of deletions sized 250-450 bp (Figure SM1). Furthermore, the detected close link between the expansion of olfactory receptor gene family and SVs (Fig. 2A) can be explained by mechanisms such as non-allelic homologous recombination or fork stalling and template switching, that can lead to expanding or contracting gene families and SVs formation [6].

The number of TRs profiled was 844,556, from which 103,730 were categorized as polymorphic (pTR). Particular to this study was the augmentation of the usually targeted TRs, the STRs (2-6 bp) [45, 72], by also including VNTRs (7-20 bp). From the total pTRs, the latter accounted for 7.02% and widened the pig TRs spectrum. Despite lower numbers as compared to the SNPs or small indels, what makes the TRs a relevant reservoir of genetic variation is their highly polymorphic nature (Table SM3). In terms of variant annotation, the TRs displayed similar characteristics to the SNPs and small indels and were predominately located in intronic, intergenic, upstream, and downstream regions (Fig. 1). Although TRs can regulate gene expression through a variety of mechanisms [25], the TRs positioned specifically in non-transcribed genomic regions could modulate gene expression via various means, such as epigenetic modification, chromatin remodeling, transcription factor binding, or alternative splicing [73]. In support of the regulatory effect of TRs on gene expression, our TRs panel based on 24 individuals was overlapping genes that are involved in biological processes (Fig. 2) related to the Regulation of transcription by RNA polymerase II (GO:0006357). Moreover, given the diverse genetic background comprising breeds such as Piétrain, Landrace, Large White, Wild boar, and Meishan, we also observed TRs in or in the proximity of genes related to Skeletal system morphogenesis (GO:0048705). TRs are found to be enriched in genes modulating body morphology [25] and thus here we emphasize on their key role in pig and breed evolution.

The landscape of genomic features and their co-localization within the founder individuals can provide useful guidelines towards the selection of genetic markers for conducting phenotype-genotype associations. From a 500 kb window-based genome wide comparison, we observe that pTRs are more co-localized with SNPs and small indels as compared to SVs (Table 2), suggesting that SNPs would be able, to a certain extent, to capture the effects of pTRs. Nonetheless, even though they are physically co-localized, the nature of TRs (i.e. multi-allelic, high mutation, and heterozygosity rate) would only allow them to be partially tagged by nearby bi-allelic SNPs as the LD pattern is constantly and rapidly changing. For that reason, if TRs are involved in quantitative trait variation, a standard SNPs based GWAS could fail to entirely capture the TRs effects. Interestingly, genomic regions with lower density rates for SNPs, small indels, SVs, and pTRs were most pronounced on SSC8 (Figure SM4) and, therefore, these regions were characterized by high levels of homozygosity. This was in agreement with findings from Gorssen et al. [27]. To mitigate this, the high density of TRs in this SSC8 region could reduce the levels of homozygosity if TRs driven variation can be built at a faster rate over generations.

In the second part of the study, we performed a GWAS to investigate the associations of SVs and TRs with four growth and carcass traits. Prior to the GWAS, we ran the imputation step following the same strategy as in our past study [22]. To accommodate the multi-allelic nature of the TRs, Beagle [10] allowed the imputation of such variants without the necessity to decompose them into bi-allelic variants. Even though this aspect can be handled by algorithmic approaches, there are no means to address the stability of TRs, but also SVs, over two generations (i.e. from F_0_ to F_2_). Therefore, variant stability over generations could be a limiting factor in imputing SVs and TRs in general, regardless of the species. Further investigations and validations to assess the behavior of SVs and TRs in imputation procedures need to be undertaken. For elucidating biological mechanisms, it can be beneficial to incorporate SVs and TRs in association studies. With a wider genomic marker spectrum, causative mutations are even more accessible and put into the right context, the cascade of molecular events leading to the variation in complex traits can be reconstructed. Apart from association studies, Chen et al. [12] showed that by adding imputed SVs to genomic prediction in dairy cattle an increase in the prediction accuracy could not be attained. However, in the same study, the authors reported that the genetic variance explained by SVs was up to 4.57% for milk yield in bulls and 3.53% for protein yield in cows. This demonstrates the existence of a small, yet potentially relevant, contribution of the SVs to the phenotypic variance. To the best of our knowledge, no study included genome-wide high density TRs in genomic prediction studies nor in association studies in livestock.

Here, the SVs and TRs based GWAS identified additional significant variants as compared to our previous SNPs and small indels based GWAS. In the current GWAS, cumulated across all traits, 56.56% of the highly significant SVs and TRs were not tagged (r^2^<0.8) by an earlier associated SNP or small indel. Among the top five genes overlapping or in the proximity of highly significant SVs and TRs (Table 3), we have identified 15 lncRNAs (long non-coding RNAs) and 1 snRNA (small nuclear RNA) gene. LncRNAs are known to be involved in different mechanisms of gene regulation and can control the expression of nearby genes by influencing their transcription [67]. In the case of the CRCL peak (SSC17, Fig. 3D), *BMP2* (bone morphogenetic protein 2) was suggested as a strong candidate in previous studies conducted on these crosses [7, 22] but also in other pig populations (Duroc × (Landrace × Yorkshire), [42]). In the latter study, the authors indicated that a SNP (rs320706814) was the main cause of the effect on carcass length. However, we could not confirm the SNP effect in our population, as this SNP was not significantly associated nor in LD with the significant SNPs, small indels, SVs, or TRs we have identified. Li et al. [42] did not exclude the option that the causative mutation could be in fact a non-SNP or non-small indel variant. Therefore, coupled with our findings, it could be hypothesized that the nearby TR-enriched lncRNAs (upstream: *ENSSSCG00000043546*, *ENSSSCG00000047884*; downstream: *ENSSSCG00000044805*) could drive the molecular mechanisms involving the *BMP2* in carcass length variation in the current F_2_ crosses. Similarly, the GWAS signal on SSC16 for CRCL was led by a lncRNA (*ENSSSCG00000050819*) together with *ISL1* (ISL LIM homeobox 1) and has not been previously reported in the AnimalQTLdb [32]. ISL1 is known to regulate pancreatic development and insulin secretion [75] and to be paired with intergenic lncRNAs [51]. The same GWAS signal was identified for BFT, supporting the existence of additional pleiotropic loci besides the one we have previously identified for BFT and CRCL on SSC7 [7]. We also found a lncRNA (*ENSSSCG00000044971*) and a snRNA (*U6*) leading the association peak on SSC7 for ADG. Both are located between *BMP5* (bone morphogenetic protein 5) and *HMGCLL1* (3-Hydroxymethyl-3- Methylglutaryl-CoA Lyase Like 1), genes that are related to lipid metabolism [1, 79] and could be regulated via the above-mentioned non-coding RNAs. The same significant genes (i.e. *ENSSSCG00000044971* and *U6*) were identified for BFT and CRCL (Fig. 4).

For ADG, we have detected a QTL on SSC16 that was not found in the previous GWAS study, yet it has been reported in a microsatellite based linkage analysis using three out of our four crosses [56]. This aspect further supports the fact that by using SNPs or small indels one is unable to capture the full range of effects affecting phenotypes. The leading GWAS signal for BFT on SSC2 was overlapping the* SHANK2* (SH3 And Multiple Ankyrin Repeat Domains 2) gene due to several highly significant TRs and to one deletion (SSC2:2,753,494; -556 bp). A similar significant signal, involving the same variants, was identified for MFR and ADG (Fig. 4). *SHANK2*, a highly polymorphic gene containing SNPs, small indels, SVs, and TRs (Table SM4) can be proposed as a relevant gene candidate as mutations in this gene have been associated with the autism spectrum disorder in humans [47]. Accordingly, the differences in ADG, BFT, and MFR phenotypes can be a consequence of pig behavioral changes related to feed intake. Likewise, for CRCL we have identified an associated gene (i.e. *ASB4*, Ankyrin repeat and SOCS box containing 4) on SSC9 that plays a key role in the control of feeding behavior and metabolic rate [43]. Further biological roles selected from already published data about the top five genes in presented in Table 3 can be found in Table 4.

**Table 4:** Summary of published data on functional biological roles of the top five gene selection

**Gene name**	**Trait**	**Published data related to top five genes selection**
*ZFAND5*	ADG	Large loss of muscle mass [41]
*AP2A2*	ADG	Regulation of lipolysis in adipose tissue [50]
*HMGCLL1*	ADG, CRCL	Ketogenesis [1]
*PRIM2*	ADG, BFT	Obesity in humans [38]
*COL21A1*	ADG, BFT, MFR, and CRCL	Body length in fish [26], directional cell migration in development in* C. elegans *[40]
*SHANK2*	ADG, BFT, MFR	Autism spectrum disorders [47]
*TNNT3*	BFT, MFR	Regulate muscle contraction, required for growth and postnatal survival [36]
*STX, MRPL16* and *CBLIF*	BFT, MFR	Residual Feed Intake in beef cattle [65]
*ISL1*	BFT, CRCL	Regulating pancreatic development and insulin secretion [75]
*PLPP7*	MFR	Muscle function, muscle growth [60]
*PRRC2B*	MFR	Growth in fish [78]
*RALGPS1* and *XKR6*	MFR	Body fat ratio [61]
*ASTN2*	MFR	Plasma triglyceride concentration [35]
*VDR*	MFR	Body fat [48]
*VTI1B*	MFR	Body weight [2]
*BTRC*	MFR	Fatty acid composition in intramuscular fat [16]
*IGFBP5*	MFR	Lipid metabolism and insulin sensitivity [74]
*AOAH*	MFR	Fat deposition in chicken [15]
*DAAM2*	CRCL	Decreased body length [53]
*NOCT*	CRCL	Susceptibility to Diet-Induced Obesity [28]
*PON2*	CRCL	Susceptibility to Diet-Induced Obesity [66]
*ASB4*	CRCL	Feeding behavior and metabolic rate [43]
*SPPL3*	CRCL	Decreased body weight [68]
*PLCB1* and *PLCB4*	CRCL	Decreased body size [39], Growth and body size [4]

The *VRTN *(vertnin) has been shown to affect vertebrae numbers and thus carcass length in pigs due to two likely causative variants: SSC7:97,614,602A>C and SSC7:97,615,879-97,615,880ins [23]. The latter is an insertion that was predominantly found in European commercial populations at high frequency (0.59, 0.65 and 0.82 in Duroc, Large White and Landrace, respectively; [76]). Although, this insertion was not present among our significant SVs, we were able to locate the variant in three founders in a homozygous state *ins/ins*, specifically in two Landrace x Large White (sample 693 and 750) and in one Large White (sample 728). The drawback was that the identification of this variant was done by one of the SV callers (i.e. manta) whereas for the imputation step only variants supported by three callers qualified. Furthermore, also on SSC7, the gene *COL21A1* (Collagen Type XXI Alpha 1 Chain) harbored the top GWAS signal, driven by an intronic TR ((TTTG)_3_/(TTTG)_5_, SSC7:29,488,854) for BFT, MFR and CRCL with a -log10 (*p*-value) of 45.40, 12.88 and 44.28, respectively (Fig. 3). The same TR is also associated with ADG (-log10 (*p*-value) =7.24) (Table 3, Fig. 4). After a conditional GWAS (Figure SM7), remaining significant peaks were observed at a lower significance only for BFT and CRCL suggesting that for ADG and MFR there was only one quantitative trait locus responsible for these traits in this region. The top genes behind the BFT signals were *DST* (dystonin), *ENSSSCG00000044091* (lncRNA), *LRFN2* (leucine rich repeat and fibronectin type III domain containing 2) whereas for CRCL we have identified only one peak in the proximity of the intronic TR region corresponding to *LRFN2*. As *DST* was already discussed as a strong candidate in our previous GWAS, we want to draw attention on *LRFN2* that could be under the influence of the nearby lncRNA (*ENSSSCG00000044091*). *LRFN2 *knockout mice exhibited autism-like behavioural abnormalities [52] and, similar to *SHANK2* and *ASB4,* could be relevant in terms of pig feeding related behaviors. Nevertheless, due to the biological roles of *COL21A1* (Table 4) and the results of the conditional GWAS, the intronic TR (SSC7:29,488,854) is recommended as a straightforward variant for further functional validation.

## Conclusion

Even though the integration of SVs and TRs in association studies is still in its infancy, this paper demonstrates the benefits of adding additional dimensions to the panel of commonly used genomic markers (SNPs and small indels). To achieve this, we deployed an efficient strategy to utilize well phenotyped and well investigated pig experimental design established in the past. Briefly, we have identified that, despite physical co-localization, SNPs or small indels do not always capture the effects of SVs and TRs on complex traits. Furthermore, we emphasize on highly significant SVs and TRs embedded or nearby lncRNAs as relevant drivers of phenotypic variation. Overall, this paper can be regarded as a valuable resource for future studies examining SVs or TRs in the context of GWAS and how these types of variation regulate gene expression and ultimately contribute to complex trait variation.

## Methods

### Data sources

#### SNPs and small Indels

Whole-genome sequence data was available for 24 founder individuals from four F_2_ pig resource populations. The founder data set was comprised of 14 Piétrain (samples: 10345, 17118, 17123, 17161, 17165, P102, P107, P108, P113, P115, P119, P128, P130 and P244), 7 crossbred Landrace x Large White (samples: 662, 690, 693, 735, 750, 756 and 771), 1 Large White (sample 728), 1 Wild boar (sample P181) and 1 Meishan individual (sample M199). The F_2_ designs under investigation were described in detail by Rückert and Bennewitz [64] and Borchers et al. [8]. In our previous study, Falker-Gieske et al. [22] provided information on the read mapping and the variant calling for short variants (i.e. SNPs and small indels < 50 bp) carried out based on the genome assembly Sscrofa 11.1 (GCA_000003025.6 provided by Swine Genome Sequencing Consortium on NCBI).

#### Structural variants and tandem repeats profiling

SVs were called with three independent variant callers: smoove, DELLY, and manta. smoove v0.2.6 [9] was used with the settings "-p 4 --genotype". DELLY v0.7.7 [62] was employed with the default settings for germline variant calling. Further, manta v1.6.0 [11] was used with default settings. A high confidence call set was generated with SURVIVOR v1.0.7 [34]. We ran the tool with a maximum distance between breakpoints of 1000 bp, a minimum number of supporting callers of 3, SV type and strands were taken into account, and the minimum SV size was set to 50 bp (SURVIVOR settings: merge input_files.txt 1000 3 1 1 0 50). These variants were filtered by a genotyping rate of 0.8 and variants with QUAL < 6000 were removed.

To screen for all tandem repeats with a motif length of 2-20 bp (comprising both STRs and short VNTRs) we employed GangSTR [54]. A pre-requisite for this tool was to set up a library of known TRs based on the reference genome. Thus for establishing this panel of repetitive regions, the repeat annotation on the reference genome was conducted using the Tandem Repeats Finder [5] with the options 2 5 17 80 10 24 1000. The initial library was filtered according to several criteria: i) size of the repeat unit 2-20 bp, ii) all overlapping TRs removed, iii) TRs located within less 50 bp of another TR removed, iv) repeat units of 2 to have minimum of five copies, v) repeat units of 3 bp to have minimum four copies and vi) repeat units > 3 bp to have at least three copies. The TRs were called in a multi-sample manner using GangSTR with the default parameters together with the bam files of our samples and the trimmed TR reference panel. The discovered TRs were then subjected to a call-level quality control filtering in which genotypes with a minimum sequence depth (DP) of 10 and with a quality score (Q) higher than 0.8 were kept. Finally, only TRs with a call rate higher than 0.8 were included in the final TRs dataset.

### Feature landscape

Each autosome was binned into successive 500 kb windows dividing the genome into 4,538 windows. The density of the features (SNPs, small indels, SVs, TRs and pTRs) was simply counted window-wise based on the starting position of the feature (e.g. the POS field in the vcf file). To investigate the co-localization of the various types of variation, we performed pairwise correlation using the Pearson’s product-moment correlation (function cor.test() under R environment, [59]). Consequently, for each pair of features a Pearson correlation coefficient* r* and the *p*-value of the test were available.

### Variant annotation, functional effect prediction, and gene enrichment analysis

To annotate variants and predict the coding effects of genetic variation (i.e. SNPs and small indels, SVs, TRs and pTR) on genes, transcripts, protein sequence, and regulatory elements, we used the SnpEff tool [14]. The database containing the genomic annotations for Sscrofa 11.1 (ENSEMBL release 99) was built and further utilized for annotation and effect prediction purposes. Based on the severity of the variant consequence, we prioritized on variation that has a high, moderate, and low impact. The gene sets enclosing these types of variants were used for over-representation analysis using the ShinyGO Gene Ontology Enrichment Analysis tool [24], targeting biological processes (BP). The aim was to determine if a set of genes shares more or fewer genes with predefined gene sets (associated with BP) than one would expect by chance. For all the gene set analyses, we used a false discovery rate (FDR) cutoff of 0.05.

### Haplotype construction and imputation

For haplotype estimation, SNPs and small indels from our previous study [22] were merged with the SVs and pTRs. Filtering procedures of SNPs and small indels have been described in detail in [22] whereas filtering of SVs and pTRs that were used in the F_0_ reference genotype panel is described in the section “Structural variants and tandem repeats profiling”. The average depth of coverage of SNPs/small indels was 21.08 and the average sequencing depth of pTRs was 27.11. Average sequencing depth for SVs could not be calculated due to the nature of the pipeline, in which SURVIVOR creates a high confidence call set from the output of three variant callers. The resulting VCF file was phased with Beagle 5.2 [10] and low coverage sequenced F_1_ individuals from the above-mentioned study were imputed with Beagle 4.0 and pedigree information. The resulting VCF was phased with Beagle 5.2 and used a reference panel for imputation of medium density (60k) chip genotyped F_2_ individuals [22]. All phasing and imputation procedures with Beagle were run with default settings. The only deviation from the imputation strategy that we employed in our previous study was the usage of Beagle 5.2 instead of Beagle 5.0 for haplotype phasing. SNPs and small indels were removed with GATK v4.1.8.1 SelectVariants [49] to produce the final SVs and TRs imputed dataset for downstream analyses.

### Genome wide association studies

GCTA v1.93.2 beta was used for single trait association analyses [77] for previously investigated phenotypes (based on SNPs and small indels) in these crosses: average daily gain, backfat thickness, meat to fat ratio, and carcass length. The phenotypes were pre-corrected for various fixed effects (e.g. stable, slaughter month) as described in [22]. Prior to the GWAS step, the multi-allelic variants were split into multiple rows (i.e. bi-allelic) using bcftools norm [17]. A mixed linear model “leave one chromosome out” (LOCO) analysis was used based on the following model y* = *a* + *xb* + *g*^*-*^ + *e*, where y* is the adjusted phenotype, *a* is the mean term, *b* is the additive effect size (fixed effect) of the candidate SV or TR to be tested for association, *x* is the SV or TR indicator variable (coded 0/1/2), *g*^*-*^ is the polygenic effect (random effect) and in case of the LOCO analysis is the accumulated effect of all SNPs except those on the chromosome where the candidate SV or TR is located. Multiple genomic relationship matrices (GRMs) were created from the F_2_ 60k SNP chip data by excluding each chromosome once. The imputed SVs and TRs with a minor allele frequency (MAF) cutoff of 1 % were used in the model together with an additional cross covariate (4 classes representing each of the 4 crosses). The choice of *p*-value significance level of marker effects was set up by the corresponding Bonferroni correction of the *p*-value of 0.05/Number of tests, where the number of tests here was the total number of SVs and TRs (54,076), therefore the -log10(*p*-value) threshold in this analysis was 6.03. Furthermore, a conditional association study was carried out. For this, top highly associated variants were included as a fixed effect in the mixed linear model framework. Manhattan plots and other figures were created in R using the package *qqman *[69] and *ggplot2 *[71].

## Supplementary Information

**Additional file 1.**

## Data Availability

The datasets used and/or analyzed during the current study are available from the corresponding author on reasonable request. The raw WGS data is available under BioProject ID PRJNA553106. The SNP array data and the phenotypes are available under https://doi.org/10.25387/g3.8287847.
