# Supplementary material for: Structural variants and tandem repeats in the founder individuals of four F2 pig crosses and implications to F2 GWAS results
Source: BMC Genomics. 2022 Sep 3;23:631. doi: 10.1186/s12864-022-08716-0 (PMC9440560; doi:10.1186/s12864-022-08716-0)
Supplement: Supplementary file 1 — Additional file 1. [file 12864_2022_8716_MOESM1_ESM.docx]

**SUPPLEMENTARY MATERIAL**

Structural variants and tandem repeats in the founder individuals of four F_2_ pig crosses and implications to F_2_ GWAS results

**Table SM1. Additional information for the founder F_0_ samples.**

| **Sample** | **ID** | **Breed** | **BioSample** | **SRA** |
| --- | --- | --- | --- | --- |
| 1 | P107 | Piétrain | SAMN12229111 | SRS5069996 |
| 2 | P244 | Piétrain | SAMN12229122 | SRS5069979 |
| 3 | P108 | Piétrain | SAMN12229128 | SRS5069973 |
| 4 | P130 | Piétrain | SAMN12229129 | SRS5069970 |
| 5 | P113 | Piétrain | SAMN12229132 | SRS5069984 |
| 6 | P128 | Piétrain | SAMN12229133 | SRS5069983 |
| 7 | P119 | Piétrain | SAMN12229134 | SRS5069982 |
| 8 | P102 | Piétrain | SAMN12229112 | SRS5069997 |
| 9 | P115 | Piétrain | SAMN12229113 | SRS5069998 |
| 10 | 17123 | Piétrain | SAMN12229114 | SRS5069999 |
| 11 | 17118 | Piétrain | SAMN12229115 | SRS5069992 |
| 12 | 17161 | Piétrain | SAMN12229116 | SRS5069993 |
| 13 | 10345 | Piétrain | SAMN12229117 | SRS5069994 |
| 14 | 17165 | Piétrain | SAMN12229118 | SRS5069995 |
| 15 | 750 | Landrace x Large White | SAMN12229119 | SRS5069990 |
| 16 | 756 | Landrace x Large White | SAMN12229120 | SRS5069991 |
| 17 | 735 | Landrace x Large White | SAMN12229121 | SRS5069978 |
| 18 | 728 | Large White | SAMN12229123 | SRS5069976 |
| 19 | 693 | Landrace x Large White | SAMN12229124 | SRS5069977 |
| 20 | 690 | Landrace x Large White | SAMN12229125 | SRS5069974 |
| 21 | 662 | Landrace x Large White | SAMN12229126 | SRS5069975 |
| 22 | 771 | Landrace x Large White | SAMN12229127 | SRS5069972 |
| 23 | P181 | Wild Boar | SAMN12229130 | SRS5069971 |
| 24 | M199 | Meishan | SAMN12229131 | SRS5069985 |

**Table SM2. Number and lengths of SVs with various sizes.**

| **Length class** | **Sum of SV length in the class** | **Percentage from total length of SV** | **Number SV in the class** | **Percentage from total number of SV** |
| --- | --- | --- | --- | --- |
| 50-1,000 | 3,831,798 | 5.63 | 11,069 | 83.85 |
| 1,000-10,000 | 5,326,261 | 7.83 | 1,582 | 11.98 |
| 10,000-50,000 | 4,223,585 | 6.21 | 164 | 1.24 |
| 50,000-100,000 | 5,516,181 | 8.11 | 79 | 0.60 |
| 100,000-500,000 | 24,873,107 | 36.56 | 108 | 0.81 |
| 500,000-1,000,000 | 24,265,340 | 35.66 | 35 | 0.26 |


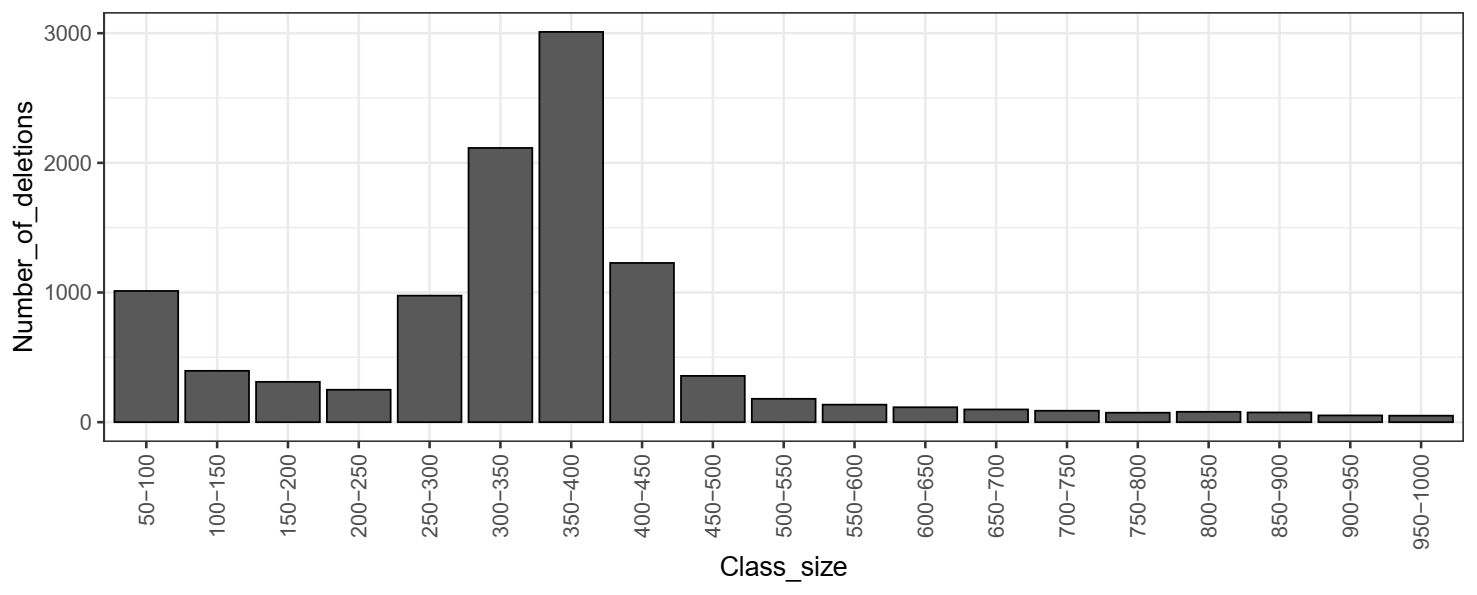


**Figure SM1. Deletion length distribution from 50 bp to 1000 bp (step 50 bp).**


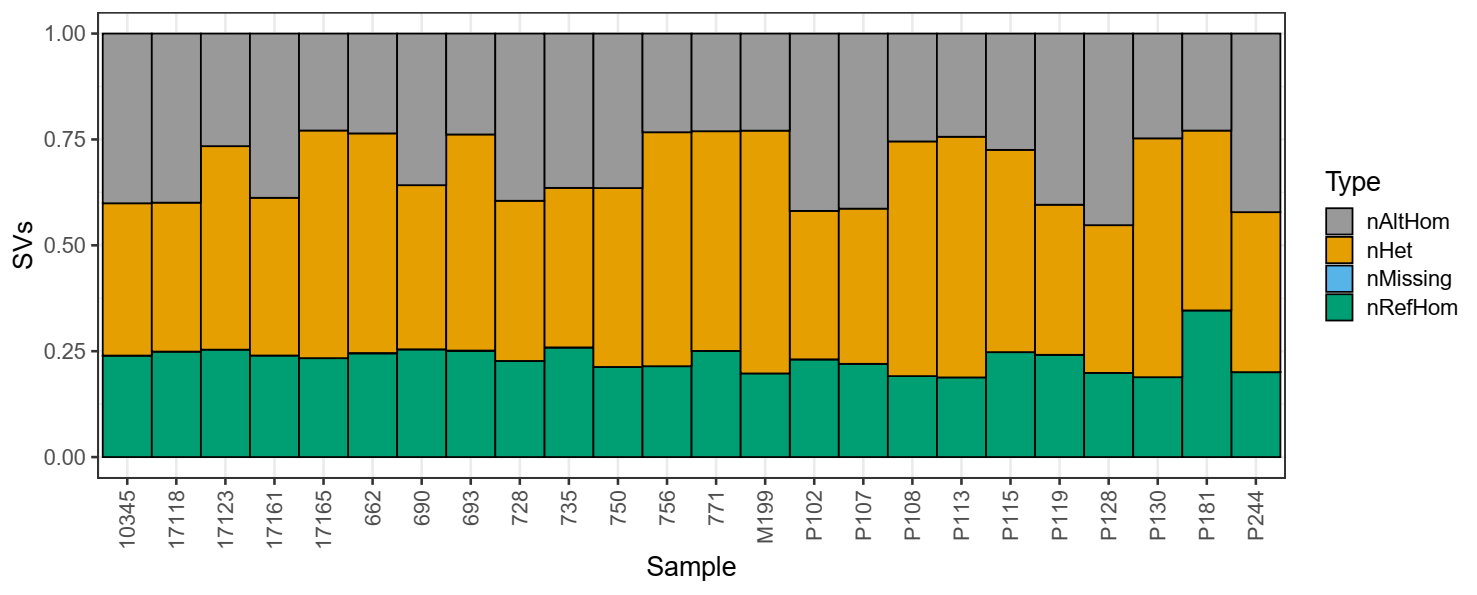


**Figure SM2**. **Individual SVs genome composition**. nMissing = missing SV genotype, nRefHom = SV that is reference homozygous, nHet SV that is heterozygous and nAltHom = SV that is alternative homozygous. 662, 690, 693, 735, 750, 756 and 771 are crossbred Large White x Landrace; 728 is Large White; 10345, 17118, 17123, 17161, 17165, P102, P107, P108, P113, P115, P119, P128, P130 and P244 are Piétrain; P181 is Wild boar; M199 is Meishan.


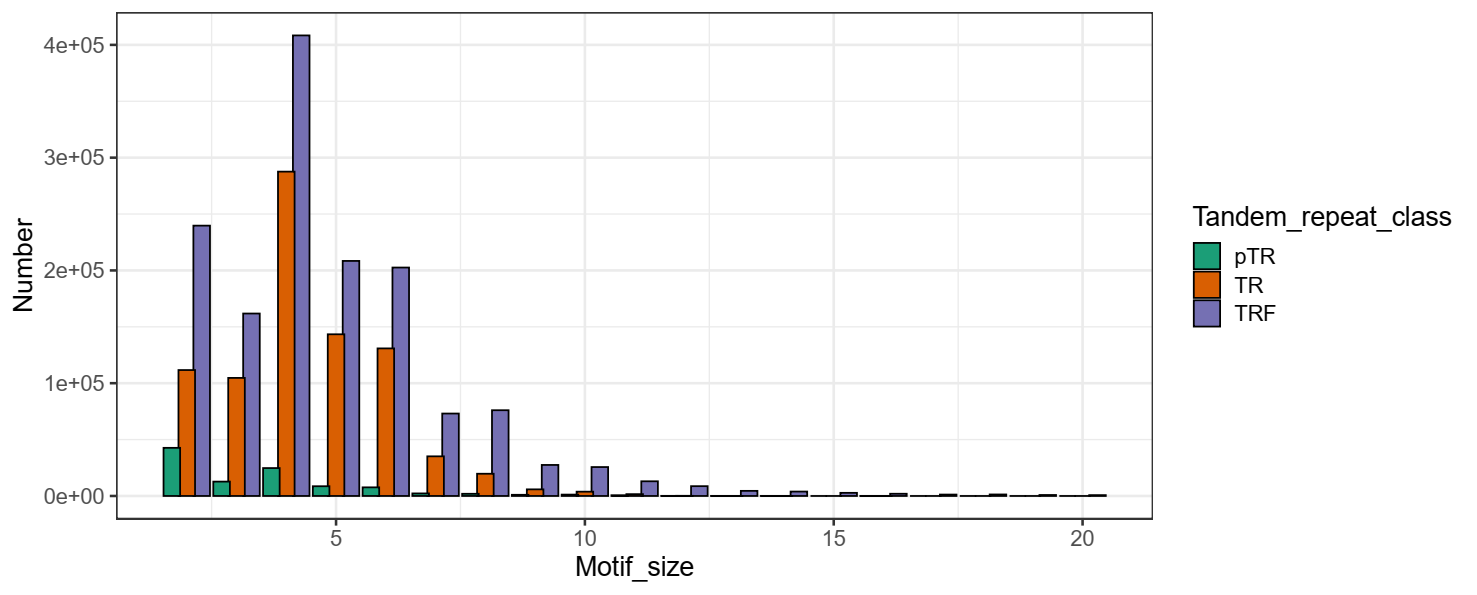


**Figure SM3. Number of tandem repeats for motifs from 2bp to 20bp.** Classes are tandem repeats in the reference genome (TRF), obtained via genotyping (TR) and, from the latter, polymorphic genotypes (pTR).

**Table SM3. Characterisation of polymorphic TR in the founder individuals based on the motif length [2-20] bp and number of alleles per motif.**

| Motif length/number of alleles | 2 | 3 | 4 | 5 | 6 | 7 | 8 | 9 | 10 | 11 | Total pTR in each motif class |
| --- | --- | --- | --- | --- | --- | --- | --- | --- | --- | --- | --- |
| 2 | 24330 | 10817 | 4546 | 1856 | 724 | 277 | 95 | 27 | 5 | 1 | 42678 |
| 3 | 9655 | 2383 | 551 | 112 | 28 | 5 | 2 | 1 | 0 | 0 | 12737 |
| 4 | 19121 | 4592 | 856 | 144 | 28 | 3 | 1 | 0 | 0 | 0 | 24745 |
| 5 | 6815 | 1564 | 227 | 28 | 4 | 0 | 0 | 0 | 0 | 0 | 8638 |
| 6 | 6193 | 1321 | 129 | 7 | 2 | 0 | 0 | 0 | 0 | 0 | 7652 |
| 7 | 1949 | 309 | 13 | 2 | 0 | 0 | 0 | 0 | 0 | 0 | 2273 |
| 8 | 1720 | 219 | 6 | 2 | 2 | 0 | 0 | 0 | 0 | 0 | 1949 |
| 9 | 958 | 83 | 2 | 0 | 0 | 0 | 0 | 0 | 0 | 0 | 1043 |
| 10 | 1152 | 64 | 1 | 0 | 0 | 0 | 0 | 0 | 0 | 0 | 1217 |
| 11 | 634 | 22 | 0 | 0 | 0 | 0 | 0 | 0 | 0 | 0 | 656 |
| 12 | 69 | 15 | 0 | 0 | 0 | 0 | 0 | 0 | 0 | 0 | 84 |
| 13 | 18 | 5 | 0 | 0 | 0 | 0 | 0 | 0 | 0 | 0 | 23 |
| 14 | 7 | 1 | 0 | 0 | 0 | 0 | 0 | 0 | 0 | 0 | 8 |
| 15 | 2 | 1 | 0 | 0 | 0 | 0 | 0 | 0 | 0 | 0 | 3 |
| 16 | 9 | 0 | 0 | 0 | 0 | 0 | 0 | 0 | 0 | 0 | 9 |
| 17 | 3 | 2 | 0 | 0 | 0 | 0 | 0 | 0 | 0 | 0 | 5 |
| 18 | 4 | 0 | 0 | 0 | 0 | 0 | 0 | 0 | 0 | 0 | 4 |
| 19 | 2 | 0 | 0 | 0 | 0 | 0 | 0 | 0 | 0 | 0 | 2 |
| 20 | 3 | 1 | 0 | 0 | 0 | 0 | 0 | 0 | 0 | 0 | 4 |
| Total pTRs in each allelic class | 72644 | 21399 | 6331 | 2151 | 788 | 285 | 98 | 28 | 5 | 1 |  |

**
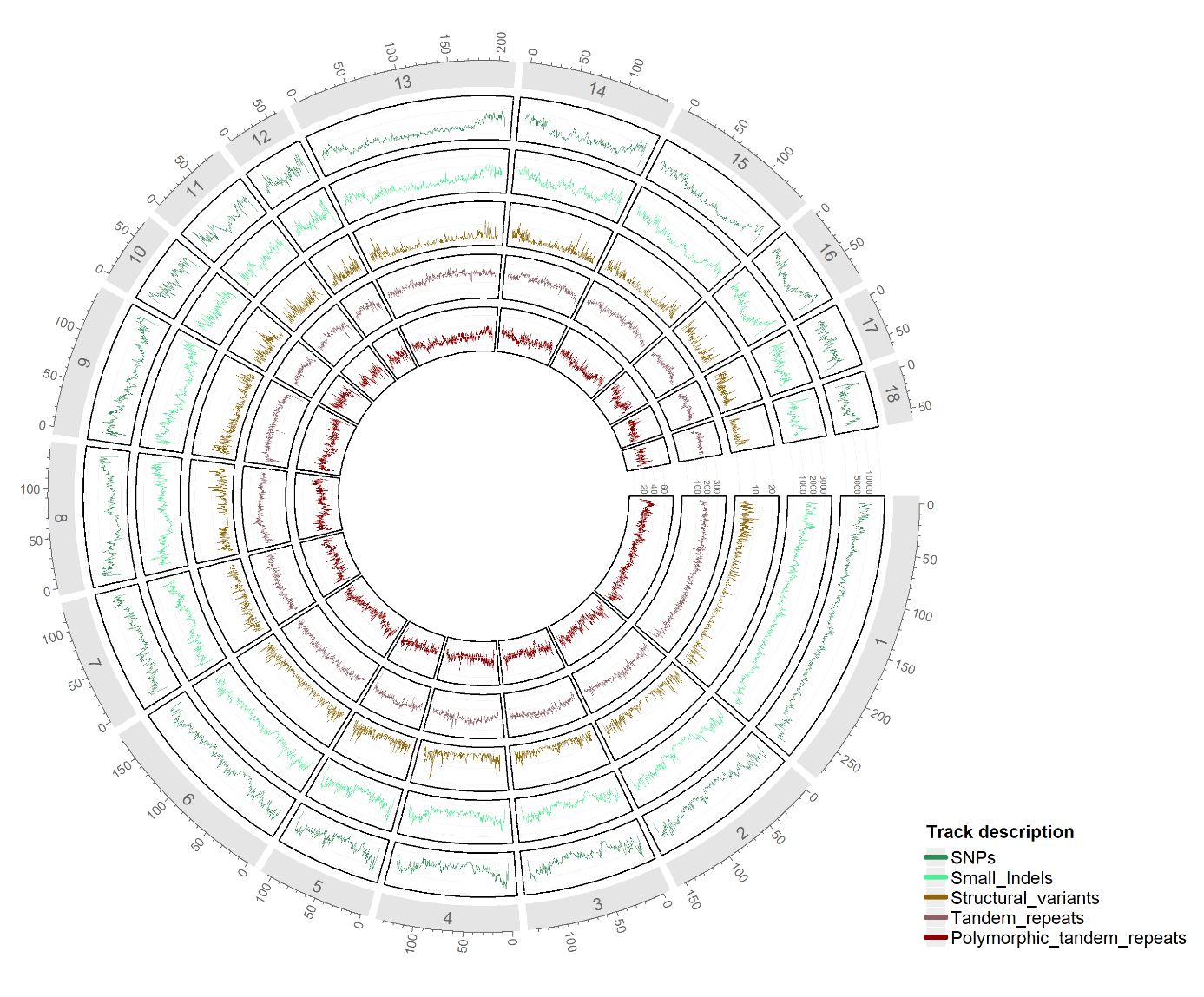
**

**Figure SM4. Chromosome wise feature density in 500 kb windows.**

**Table SM4. Overlapping genes among the gene sets (n=4) containing high, moderate and low impact variants (SNPs, small indels, SVs and TRs).**

| **Gene stable ID** | **Gene name** | **Chr** | **Gene start (bp)** | **Gene end (bp)** | **Gene description** |
| --- | --- | --- | --- | --- | --- |
| ENSSSCG00000004511 |  | 1 | 99417231 | 99809899 | myosin VB |
| ENSSSCG00000033043 | SHANK2 | 2 | 2503181 | 3016433 | SH3 and multiple ankyrin repeat domains 2 |
| ENSSSCG00000013113 | TMEM132A | 2 | 10814306 | 10824894 | transmembrane protein 132A |
| ENSSSCG00000013347 | PRMT3 | 2 | 38884361 | 39032916 | protein arginine methyltransferase 3 |
| ENSSSCG00000027371 | PCDHGA4 | 2 | 142983077 | 143156558 | protocadherin gamma subfamily A, 4 |
| ENSSSCG00000008549 | SLC4A1AP | 3 | 111453816 | 111531625 | solute carrier family 4 member 1 adaptor protein |
| ENSSSCG00000008591 | ATAD2B | 3 | 114722321 | 114846644 | ATPase family AAA domain containing 2B |
| ENSSSCG00000006346 | ATF6 | 4 | 88591280 | 88818372 | activating transcription factor 6 |
| ENSSSCG00000006731 | VTCN1 | 4 | 103412571 | 103584684 | V-set domain containing T cell activation inhibitor 1 |
| ENSSSCG00000038825 |  | 6 | 56453731 | 56592351 |  |
| ENSSSCG00000029231 |  | 6 | 58707761 | 58750154 |  |
| ENSSSCG00000002446 |  | 7 | 113608783 | 113645784 | ataxin 3 |
| ENSSSCG00000015556 | LAMC2 | 9 | 124435807 | 124495354 | laminin subunit gamma 2 |
| ENSSSCG00000010896 | ASPM | 10 | 19975189 | 20039623 | assembly factor for spindle microtubules |
| ENSSSCG00000025996 | MTUS2 | 11 | 5953753 | 6532959 | microtubule associated scaffold protein 2 |
| ENSSSCG00000034348 | NBEA | 11 | 11084534 | 11722852 | neurobeachin |
| ENSSSCG00000035852 | CSNK1D | 12 | 768865 | 796532 | casein kinase 1 delta |
| ENSSSCG00000029944 | FASN | 12 | 920507 | 937559 | fatty acid synthase |
| ENSSSCG00000022659 | CLTC | 12 | 35849136 | 35926023 | clathrin heavy chain |
| ENSSSCG00000017907 | PFN1 | 12 | 51961696 | 51973660 | profilin 1 |
| ENSSSCG00000032472 | SLC25A11 | 12 | 51970805 | 51975548 | solute carrier family 25 member 11 |
| ENSSSCG00000012071 |  | 13 | 203325350 | 203372361 |  |
| ENSSSCG00000015843 | UBXN8 | 15 | 54339563 | 54367605 | UBX domain protein 8 |
| ENSSSCG00000016284 |  | 15 | 132918912 | 132980228 |  |
| ENSSSCG00000022915 | DDX27 | 17 | 50903198 | 50924813 | DEAD-box helicase 27 |

**
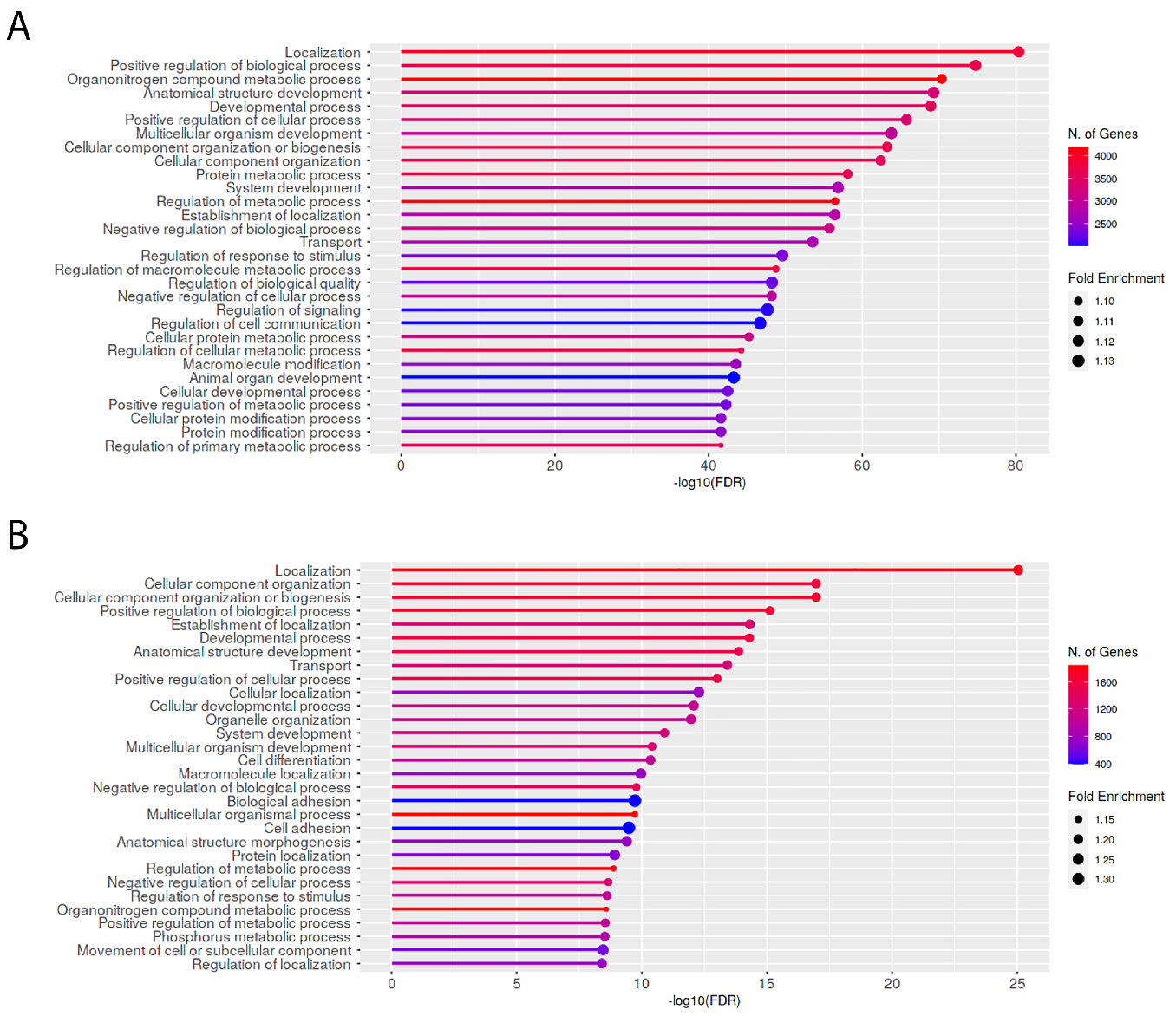
**

**Figure SM5. Gene enrichment analysis**. A. Top 30 enriched GO Biological Processes for SNPs overlapping genes (n= 19,483); B. Top 30 enriched GO Biological Processes for small indels overlapping genes (n= 8,512).


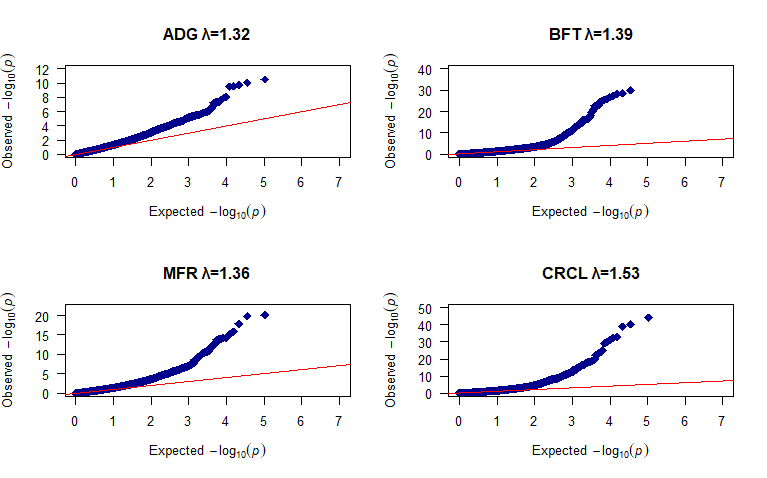


**Figure SM6. QQ plots of each GWAS.** Genomic inflation factor is λ.


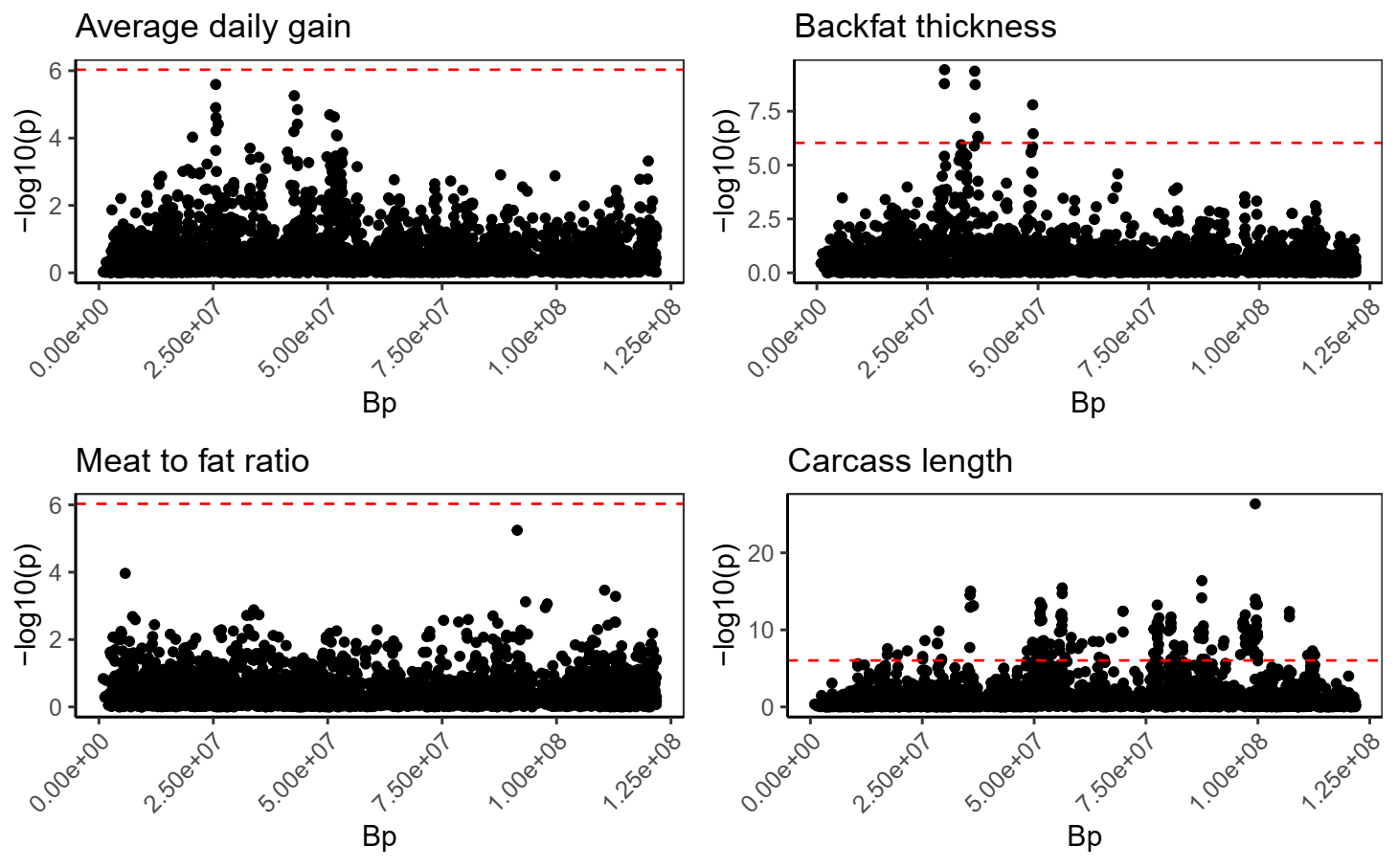


**Figure SM7. Manhattan plot of the conditional genome wide association studies on SSC7 for average daily gain, backfat thickness, meat to fat ratio and carcass length.** The top significant TR ((TTTG)_3_/(TTTG)_5_, SSC7:29,488,854) was added as a fixed effect in the LOCO mixed linear model. The genome-wide significant threshold is given by –log10 (0.05/54,075).

**
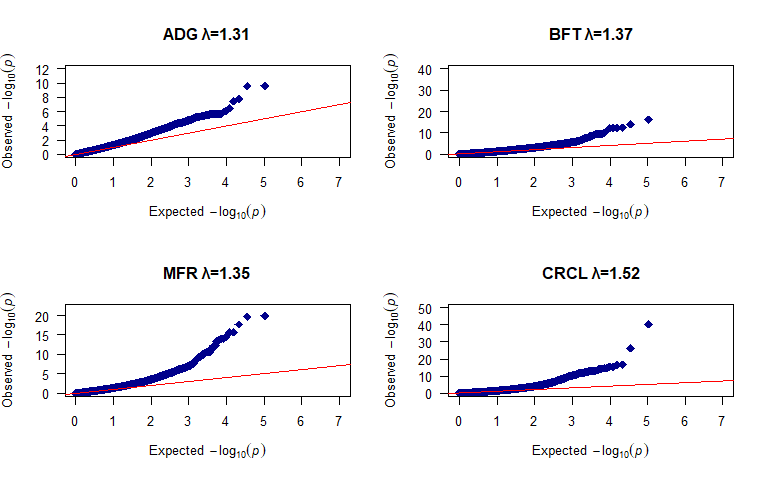
**

**Figure SM8. QQ plots of each conditional GWAS.** Genomic inflation factor is λ.
